# Olfactory dysfunction in chronic stroke patients

**DOI:** 10.1186/s12883-015-0463-5

**Published:** 2015-10-12

**Authors:** Eike Wehling, Halvor Naess, Daniel Wollschlaeger, Hakon Hofstad, Annika Bramerson, Mats Bende, Steven Nordin

**Affiliations:** Department of Physical Medicine and Rehabilitation, Haukeland University Hospital, Bergen, Norway; Department of Biological and Medical Psychology, University of Bergen, Bergen, Norway; Kavli Centre for Aging and Dementia Research, Haraldsplass Hospital, Bergen, Norway; Department of Neurology, Haukeland University Hospital, Bergen, Norway; Centre for Age-related Medicine, Stavanger University Hospital, Stavanger, Norway; Institute of Clinical Medicine, University of Bergen, Bergen, Norway; Institute for Medical Statistics, Epidemiology and Informatics, University Medical Center, Johannes-Gutenberg-University, Mainz, Germany; Department of Otorhinolaryngology, Central Hospital, Skövde, Sweden; Department of Psychology, University of Umeå, Umeå, Sweden

**Keywords:** Olfaction, Stroke, Odor identification

## Abstract

**Background:**

The aim of the study was to investigate odor identification performance in patients one year after hospital admittance due to stroke. Predictors for olfactory dysfunction were investigated as well as self-reported olfactory function and pleasantness of olfactory items.

**Methods:**

A 1-year prospective study was performed. Stroke location, classification and comorbidities were registered at hospital admission. One year after admission, olfactory function was assessed using standardized olfactory methods (screening for loss of detection sensitivity and an odor identification test). A group of matched controls was derived from a population-based study to compare odor identification performance between groups. Patients were asked for their personal judgment regarding their olfactory function and pleasantness of odorous items. In addition, global cognitive function and symptoms of depression were assessed.

**Results:**

A total of 78 patients were enrolled (46 males, 32 females; mean age 68 years) of which 28.2 % exhibited reduced olfactory function (hyposmia) and 15.4 % exhibited loss of olfactory function (10.3 % functional anosmia, 5.1 % complete anosmia). Patients showed significantly lower olfactory performance compared to age- and sex-mated matched controls. Predictors of impaired olfactory function were age and NIHSS score. Self-reports indicated no significant differences between patients with normal olfactory function and those with reduced function. Yet, patients having an olfactory dysfunction rated odorous items as significantly less pleasant compared to patients without dysfunction.

**Conclusions:**

Olfactory dysfunction seems to occur frequently after stoke even one year after initial admission. The deficits seem to relate to hyposmia and functional anosmia, and less to a complete loss of smell sensitivity.

## Background

The sense of smell provides us with crucial information about the environment and contributes to important aspects of life. Lately, interest in olfactory dysfunction has increased due to findings indicating that olfactory dysfunction is associated with increased risk of mortality [1], degenerative disease such as Alzheimer’s disease and Parkinson’s disease [2, 3], as well as due to the general appreciation of olfaction being important for quality of life. Olfactory dysfunction can result in altered food enjoyment, poor appetite, difficulties preparing food, inability to detect hazardous conditions (e.g., gas, smoke and spoiled food), lack of understanding of others and social isolation, depression and mood changes [4, 5].

Although a rather large number of medical conditions are well documented regarding their impact on olfaction [6], only a few studies have investigated olfactory function in stroke patients. Population-based studies that have estimated the risk of olfactory dysfunction in the aging segment of the population provide ambiguous results regarding stroke. Whereas both Murphy et al. [7] and Karpa et al. [8] showed that stroke was a risk factor for olfactory dysfunction, Schubert et al. [9] and Landis et al. [10] could not support these findings. The remaining available literature on stroke and olfaction includes case studies. For example, these studies show that stroke patients do not have a complete loss of olfactory function, but rather experience unpleasant sensation of odors [11–16]. Cecchini et al. [17] reported slightly reduced olfactory function in two out of the three patients they investigated.

One aim of this study was to investigate the ability to identify odors in chronic stroke patients. This was studied by comparing patients’ performance on a common clinical measure of olfactory function [18–20] with an age- and sex-matched sample of referents from a population-based study. The stroke patients and referents were compared with respect to performance score on odor identification as well as distribution of olfactory diagnosis. This included normosmia (normal olfaction), hyposmia (reduced olfactory function) and functional anosmia (significantly impaired olfaction, including both total loss and minimal residual perception) [21]. A second aim was to investigate predictors of odor identification performance. This comprised functional effects of stroke at the time of admission and during follow-up. A third aim was to compare patients being classified as normosmic versus hyposmic/anosmic with respect to self-reported olfactory functioning and odor pleasantness ratings.

## Methods

### Stroke patients

The sample of stroke patients consisted of patients admitted to the Stroke Unit at the Department of Neurology, Haukeland University Hospital (HUH), Bergen, Norway in 2009–2012. These patients were part of a larger project on rehabilitation. Inclusion criteria comprised patients living at home at the time of admission, a verification of stroke by magnet resonance imaging (MRI) or computer tomography (CT), an NIHSS score between 2 and 26, and a score ≥2 on the modified Rankin Scale. Patients had to be awake and able to consent. If the patient was not capable of giving his or her consent it was given by a next-of-kin. There was no age-limit. Exclusion criteria were serious psychiatric disorder, alcohol or substance abuse, serious conditions interfering with the subsequent rehabilitation process, and insufficient knowledge of the Norwegian language (pre-stroke). At initial hospital admission, the patients underwent a comprehensive neurological examination to establish a diagnosis of stroke, clinical syndrome, pathological, and etiological subtype of stroke and neurological deficits, as assessed by the NIHSS [22], and MRI or CT. One year after initial admission, the patients were invited to return for a comprehensive neuropsychological assessment including a battery of cognitive tests, questionnaires, and assessment of olfactory function. All patients gave written informed consent. In total, 78 patients (*n* = 73 with cerebral infarction, *n* = 5 with cerebral hemorrhage) were initially screened for anosmia regarding odor detection sensitivity with a simplified version (for details, see [23]) of the Connecticut Chemosensory Clinical Research Center Threshold Test [24]. Four patients were evaluated as anosmic, making the subsequent test of odor identification meaningless. The remaining 74 patients underwent assessment with the odor identification test. Patient characteristics are shown in Table 1.

**Table 1:** Baseline characteristics of all patients according to the protocol at admission and olfactory status at follow-up

	Total n	Normosmic	Hyposmic	Anosmic
Total, %	78	44 (56.4 %)	22 (28.2 %)	12 (15.4 %)
Male	46	27 (58.7 %)	11 (23.9 %)	8 (17.4 %)
Stroke type, ischemic	73	43 (58.9 %)	19 (26.0 %)	11 (15.1 %)
NIHSS score on admission*		3.9 (3.8)	7.3 (4.3)	6.0 (5.0)
Diabetes	9	4 (44.4 %)	2 (22.2 %)	2 (22.2 %)
Myocardial infarction	9	4 (44.4 %)	5 (55.6 %)	–
Angina pectoris	9	4 (44.4 %)	1 (11.1 %)	4 (44.4 %)
Peripheral artery disease	3	1 (33.3 %)	2 (66.7 %)	–
Leukoaraiosis	22	13 (59.1 %)	7 (31.8 %)	2 (9.1 %)
Hypertension	39	21 (53.8 %)	10 (25.6 %)	8 (20.5 %)
Earlier stroke	9	6 (66.7 %)	2 (22.2 %)	1 (11.1 %)
Earlier transient ischemic attack (TIA)	5	2 (40 %)	2 (40 %)	1 (20 %)
Smoking on admission	18	12 (66.7 %)	5 (27.8 %)	1 (5.6 %)
1 year follow up				
Age ≥60 years at follow-up	57	33 (57.9 %)	15 (26.3 %)	9 (15.8 %)
MMSE*		28.2 (2.1)	27.7 (2.5)	27.8 (2.5)
HADS D*		2.7 (1.9)	2.9 (2.6)	3.4 (2.5)
Smoking at follow up	9	6 (66.7 %)	3 (33.3 %)	–
Problems breathing through nose	2	1 (50 %)	1 (50 %)	–
Frequent sinusitis	2	1 (50 %)	1 (50 %)	–

*M(SD)

On admission, clinical stroke subtype was classified according to the Oxfordshire Community Stroke Project (OCSP) [25]. This included partial anterior circulation infarct (PACI, *n* = 37), posterior circulation infarct (POCI, *n* = 12), total anterior circulation infarct (TACI, *n* = 4), and lacunar circulation infarct (LACI, *n* = 20). According to the protocol at the HUH, the following risk factors were registered on admittance: hypertension (*n* = 37), smoking at admission (*n* = 18), diabetes mellitus (*n* = 8), myocardial infarction (*n* = 9), angina pectoris (*n* = 7), and peripheral artery disease (*n* = 3) (Table 2).

Hypertension was defined as prior use of antihypertensive medication. Diabetes mellitus was considered present if the patient was on glucose-lowering diet or medication. Angina pectoris, myocardial infarction and peripheral artery disease were considered present if diagnosed by a physician any time before stroke onset. Atrial fibrillation required ECG confirmation any time prior to stroke onset or during the hospital stay. A history of prior stroke was registered. In 17.9 % (*n* = 14) of the sample, the present stroke was a recurrent vascular event; 11.5 % were registered with an earlier stroke, 6 % with a transient ischemic attack (TIA). The etiology of ischemic stroke was determined by TOAST (the Trial of Org 10172 in Acute Stroke Treatment classification), including large artery atherosclerosis (*n* = 11), cardioembolism (*n* = 21), small vessel occlusion (*n* = 14), other etiology (*n* = 1), and undetermined etiology (*n* = 26) (Table 2). NIHSS scores ranged from 0 to 18 (Median 4), indicating that the patients, on average, were slightly to moderately affected by the stroke. By the time of admission, all patients were living at home independently, and one received help from the district nursing office.

**Table 2:** Distribution of stroke causes (TOAST criteria) and stroke location (Oxfordshire Community Stroke Project Criteria (OCPS)

Criteria	Total n	Normosmic	Hyposmic	Anosmic
TOAST criteria				
Large artery atherosclerosis	11	7	2	2
Cardioembolism	21	14	4	3
Small vessel occlusion	14	7	4	3
Stroke of other determined etiology	1	1	0	0
Stroke of undetermined etiology	26	11	11	4
OCPS criteria				
PACI	37	18	11	8
POCI	12	9	2	1
TACI	4	2	1	1
LACI	20	11	7	2

A population-based sample of referents (*n* = 148) were semi-randomly selected from the Skövde Population-Based Study [26] for comparison with the sample of stroke patients who underwent assessment with the Scandinavian Odor Identification Test (SOIT) [27]. Each stroke patient was matched subject-by-subject for age (±1 year) and sex to two referents. Both samples had a mean age of 67.0 (SD = 12.5) years. The patients consisted of 44 men and 30 women, and the referents of 88 men and 60 women.

The main project was approved by the Regional Committee for Research Ethics of Western Norway. The studies were performed according to the Declaration of Helsinki on guidelines for biomedical research involving human subjects (World Medical Association, 2013).

### Scandinavian odor identification test

The SOIT was used to objectively assess olfactory function; a test which has adequate psychometric properties [27] and is valid for use in Norwegian samples [28]. The test includes the sixteen odorants pine needle, peppermint, juniper berry, violet, anise, clove, vanilla, almond (bitter), orange, cinnamon, lemon, lilac, vinegar, tar, ammonia, and apple. Ammonia (1.0 M), tar, and vinegar were natural products while the other odorants were natural oils (Stockholm Ether & Essence Manufactory, Stockholm, Sweden). The odorant was injected into a tampon filled to saturation and placed into an opaque, 80 ml glass jar, sealed with a teflon lock. The stimuli were presented birhinally 1–5 cm under the participants’ nose. A card with four written response alternatives was placed in front of the participant with the instruction to choose the item that most appropriately identified the odor, with a forced-choice procedure. To avoid adaptation, each stimulus was presented no longer than 3–4 s. No time restrictions were given for the participants to make their choice. The inter-stimulus interval was about 20 s to avoid significant effects of adaptation [29]. Testing was conducted in a well-ventilated room without background odor. For analyses, the number of correctly identified odors was used. Based on this number, the participants were categorized as either normosmic, hyposmic, or functionally anosmic according to the age- and sex-dependent normative data for the SOIT [27].

### Self-reported olfactory function and pleasantness ratings

At follow-up, the participants initially underwent a short questionnaire regarding factors with potential effect on olfactory functioning (current and past smoking, problems breathing through nose, frequent sinusitis). This was followed by a question regarding self-reported olfactory functioning “How would you estimate your sense of smell on a scale from 1–5 (1 = quite poor, 5 = excellent)?” After presenting each odor item of the SOIT, the participant was asked to rate the pleasantness of that odor on a scale ranging from 1 (not pleasant) to 5 (very pleasant). Ratings were averaged over all 16 items for analyses.

### Cognitive function and depression

Global cognitive function was assessed with the Mini-Mental State Examination (MMSE), which is a brief 30-point questionnaire sampling various cognitive functions such as arithmetic, episodic memory, orientation and language skills [30]. The scale ranges from 0 to 30 (high score representing high level of cognitive function). The depression subscale of the Hospital Anxiety and Depression Scale (HADS) [31] was used to quantify degree of depression. The subscale has seven items (e.g., “I have lost interest in my appearance”), asking the respondent to answer the questions according to the feelings of the past week. The scale ranges from 0 to 21 (high score representing high anxiety/depression level).

### Statistical analyses

Mean scores on the SOIT were compared between the group of stroke patients and referents with paired t-tests averaging the SOIT performance scores from the two referents per patient. The distributions across olfactory diagnoses were compared between groups using the McNemar Bowker Test, after first averaging the SOIT performance scores from the two referents and subsequently categorizing them based on the normative data [27]. Pearson correlation coefficients were computed between, on the one hand, SOIT score and on the other hand, age, the score of the NIHSS, MMSE and HADS depression scale. T-tests were used to analyze sex-differences on SOIT score in stroke patients. Statistical predictors of SOIT score were tested in multiple linear regression analyses including age, NIHSS score, score on the depression subscale of the HADS, and MMSE score. Variables in this analysis were selected when they reached the moderately strict α-level of 0.10, for the remaining analyses, the α-level was set at 0.05. The analyses were performed using SPSS software version 21.

## Results

### Performance on odor identification

Mean (SD) score on the SOIT was 11.0 (2.6) for the stroke patients, and 12.6 (2.2) for the referent pairs, which differed significantly (*t* (72) = 4.67, *p* <0.001). Based on these scores and normative data [27], 40.5 % (*n* = 30) of the stroke patients and 23.0 % of the referent pairs had an olfactory dysfunction (hyposmia or functional anosmia). More specifically, 59.5 % (*n* = 44) of the stroke patients were normosmic, 29.7 % (*n* = 22) were hyposmic, and 10.8 % (*n* = 8) were functionally anosmic. Corresponding percentages for the referent pairs were 83.7 % (*n* = 62), 14.9 % (*n* = 11) and 1.4 % (*n* = 1), respectively. The distributions across olfactory diagnoses differed significantly between the two groups (McNemar Bowker df (3) = 13.62, *p* <0.004). Thus, compared to the referents, the stroke patients had significantly lower SOIT scores, and consisted to a significantly larger extent of persons with hyposmia and functional anosmia.

Since the group of stroke patients included a number of patients for whom an earlier vascular event (stroke or TIA) had been registered, analyses were re-run, excluding those 12 patients and their respective 24 matched controls. The differences regarding SOIT score and distribution across olfactory diagnoses remained significant (*t* (60) = 4.13, *p* <0.001 and McNemar Bowker df (3) = 10.02, *p* <0.02, respectively.

### Predictors of olfactory dysfunction

Pearson correlation analyses indicated that age (*r* = −0.28, *p* <0.02) and NIHSS score (*r* = −0.35, *p* <0.02) correlated significantly with SOIT score, whereas MMSE score of global cognitive function (*r* = 0.2, *p* <0.09) and score on the HADS depression scale (*r* = 0.23, *p* <0.06) showed only strong tendencies of such correlations. No sex differences regarding SOIT scores were found (*t* (72) = 0.87, *p* >0.38). Results from multiple regression analyses with SOIT as dependent variable are shown in Table 3. Age and NIHSS were found to be significant predictors, whereas neither MMSE score nor HADS depression score were, when controlled for age and NIHSS score.

**Table 3:** Results of multiple hierarchical regression analyses

Predictor	B	SE B	Incr R^2^	Cum R^2^	β	p	95% CI lower bound	95% CI upper bound
Age	−0.058	0.023	0.081	0.081	−0.284	0.014	−0.105	−0.012
Age	−0.051	0.023	0.081	0.081	−0.248	0.025	−0.095	−0.007
NIHSS	−0.184	0.062	0.101	0.182	−0.321	0.004	−0.308	−0.061
Age	−0.048	0.024	0.081	0.081	−0.235	0.048	−0.096	0.00
NIHSS	−0.180	0.064	0.101	0.182	−0.313	0.006	−0.308	−0.053
MMSE	0.041	0.131	0.001	0.184	0.037	0.754	−0.220	0.302

### Self-reported olfactory functioning and pleasantness judgments

Comparison of means for self-reported olfactory function in normosmic (M = 3.72 (SD 0.97) vs. hyposmic/anosmic stroke patients (M = 3.57 (SD 0.99) revealed no significant difference regarding self-reported olfactory function (*t* (57) = 0.521, *p* = 0.604). Average mean (SD) ratings for odor pleasantness was 2.94 (0.45) for the normosmic stroke patients, and 2.52 (0.54) for the hyposmic/anosmic patients. This difference was significant (*t* (72) = 3.12, *p* = 0.003).

## Discussion

In summary, the findings demonstrate olfactory dysfunction in a significantly larger proportion of chronic stroke patients compared to a group of matched controls. Moreover, we found that age and NIHSS score (on admission) were significant predictors for olfactory dysfunction. Self-reported olfactory function did not differ between stroke patients with and without olfactory dysfunction. Yet, average pleasantness ratings differed significantly, indicating that patients with reduced olfactory function, on average, perceived olfactory items as less pleasant compared to patients with normal olfactory status.

The results indicating that olfactory dysfunction occurs as frequent as in 43 % in chronic stroke patients even one year after initial admission are notable and new. It has to be acknowledged that four patients in our study completely lacked ability to perceive odor (due to the initial screening procedure). This number is in accordance with findings from population-based studies, reporting approximately 5 % to be unable to smell [21, 26]. Additionally, another 10.3 % of the patients showed functional anosmia, based on results from the odor identification test. It should be emphasized that the high correlation between odor detection sensitivity and identification ability validates the use of identification tasks to assess hyposmia and anosmia [32]. Individuals with functional anosmia do indeed also experience impact on quality of life [5]. Thus, the frequent occurrence of olfactory dysfunction in patients stresses that clinicians should be aware of this issue in clinical routines, though maybe not in the acute phase as focus lies on other medical issues.

Our findings, indicating age to be a significant predictor for olfactory performance, are in accordance with the well-established literature [18, 28, 33]. In addition, NIHSS score remained a significant predictor even when controlling for age. The current findings extend the existing studies addressing the long-term consequences of stroke. The, NIHSS has been shown to be a predictor for stroke outcome and there is evidence showing that it may be associated with the development of vascular cognitive impairment (VCI) and eventually a diagnosis of dementia [34, 35]. Olfactory dysfunction is a well-established marker for dementia [3, 36] and is even known to occur in individuals at risk for dementia who demonstrate mild cognitive impairment [37, 38]. However, findings on VCI and olfactory dysfunction are still scarce. It has been shown that patients with vascular dementia perform below normative performance in olfactory tests [39]. Nonetheless, when comparing patients with vascular dementia and patients with AD, findings are mixed in that Gray et al. [39] report a similar degree of olfactory impairment in both patient groups, whereas Duff et al. [40] have shown lower performance in AD patients. In our sample, none of the patients had received a diagnosis of dementia at the time of assessment, although we cannot rule out that some patients may have been on a path towards decline. Moreover, the results indicate only a tendency of an association between global cognitive function (MMSE) and odor identification performance. Odor identification draws on memory functions [41], but processing speed, language proficiency and reasoning have also shown to be involved [42–44]. Cognitive changes in VCI are variable depending on the lesion location [35], plausible explanations for finding a trend only may be that the sample was too heterogeneous to detect possible associations. Additional, screening instruments for dementia such as the MMSE, which was primarily developed for assessing cognitive deficits associated with AD, often do not detect those subtle deficits as seen in VCI [35]. Yet, since up to 50 % of individuals with VCI develop dementia in a five-year period [45], the current findings underline the urgent need for more knowledge.

The finding indicating no differences for self-reported olfactory functioning between normosmic patients and those with an olfactory dysfunction are not surprising since it has repeatedly been shown that self-reports are unreliable predictors of olfactory status [28, 46, 47]. It has been discussed that due to the subtle progression of decline a certain threshold must be reached before loss becomes noticeable [37]. The present finding of unawareness of olfactory dysfunction in stroke patients underlines the need for clinicians to objectively assess olfactory function in this patient population.

It is notable that those patients with an olfactory dysfunction perceive the odors as less pleasant than normosmic patients. This is in accordance with case studies which repeatedly have reported qualitative smell and taste disturbances [11–14, 16]. These reports vary regarding the duration of the disturbances, varying between weeks and years. Our results showing that dysfunction may occur even a year after injury underline a prolonged course. Additional, existing findings have commonly stressed that the patients had not completely lost their ability to smell, but that change in perceptual quality of the odorous stimuli was a serious problem as it resulted in changes in diet and eventually weight loss [11, 16]. Our results indicate a clear trend between symptoms of depression and odor identification performance. Signs of depression are a known consequence in patients with smell loss [48]. Croy et al. [5] suggested two potential links between depression and olfactory disorders: One arising through reduced quality of life in functions depending on olfaction (eating, social communication, environmental hazards etc.) and thus indirectly leading to depression, and another directly related to brain function in that olfactory input from the olfactory bulb via amygdala and limbic system is changed. Possible consequences for our patients have to remain speculative. Yet, two recent studies have stressed a possible link between nutritional status and stroke. Paquereau et al. [49] found malnutrition to be associated with changed food intake and food preferences in 50 % of the stroke patients although their study did not include olfactory assessment. Aliani et al. [50] concluded that olfactory dysfunction in the elderly and patients with dementia can lead to dietary restrictions and thus negative implications on nutrition and overall health.

The small number of patients in this study limits the generalizability of our findings. Additionally, small cell frequencies prevented us from analyzing relationships between olfactory dysfunction and stroke classifications (i.t. OCPS/TOAST) or other medical factors occurring in patients. We included all available patients, and based on MMSE score and NIHSS (baseline) it is assumed that the patients included had rather mild to moderate impact of the stroke. Thus, olfactory dysfunction may rather be under-estimated in this sample since patients not returning after one year may have even poorer functioning than those included here. Lastly, we did not register drugs patients were taking at the time of follow-up assessment, although drug-induced olfactory (and even more taste) dysfunction is well-known [21]. Yet, although drug-induced olfactory dysfunction may be reversible, this may not always be possible in these patients.

## Conclusions

Results from the present study suggest that olfactory disorders occur frequently even in mild to moderate chronic stroke patients. The deficit seems to be related to hyposmia and functional anosmia, and less to a complete loss of smell. The findings strongly suggest the inclusion of olfactory assessment in clinical routine. Furthermore, there is urgent need to investigate long-term impact on olfactory changes in stroke patients regarding olfactory changes in VCI and dementia and the importance of olfaction for flavor and quality of life in general.
