# Erratum to: Olfactory dysfunction in chronic stroke patients

**DOI:** 10.1186/s12883-015-0489-8

**Published:** 2015-11-19

**Authors:** Eike Wehling, Halvor Naess, Daniel Wollschlaeger, Håkon Hofstad, Annika Bramerson, Mats Bende, Steven Nordin

**Affiliations:** Department of Physical Medicine and Rehabilitation, Haukeland University Hospital, Bergen, Norway; Department of Biological and Medical Psychology, University of Bergen, Bergen, Norway; Kavli Centre for Aging and Dementia Research, Haraldsplass Hospital, Bergen, Norway; Department of Neurology, Haukeland University Hospital, Bergen, Norway; Centre for Age-related Medicine, Stavanger University Hospital, Stavanger, Norway; Institute of Clinical Medicine, University of Bergen, Bergen, Norway; Institute for Medical Statistics, Epidemiology and Informatics, University Medical Center, JohannesGutenberg-University, Mainz, Germany; Department of Otorhinolaryngology, Central Hospital, Skövde, Sweden; Department of Psychology, University of Umeå, Umeå, Sweden

## Erratum

Unfortunately, the original version of this article [1] contained an error in the author list.

Håkon Hofstad was incorrectly listed as ‘Hakon Hofstad’. The original article has now been updated to reflect the correct author name.

## References

[1] Wehling E, Naess H, Wollschlaeger D, Hofstad H, Bramerson A, Bende M, et al. Olfactory dysfunction in chronic stroke patients. BMC Neurol. 2015;15:199. DOI 10.1186/s12883-015-0463-5. 10.1186/s12883-015-0463-5 PMC4604071 26459234

